# Mammary epithelial polarity and macrophage infiltration

**DOI:** 10.14800/Macrophage.1521

**Published:** 2017-03-13

**Authors:** Ren Xu

**Affiliations:** 1 Markey Cancer Center, University of Kentucky, Lexington, KY 40536, USA; 2 Department of Pharmacology and Nutritional Sciences, University of Kentucky, Lexington, KY 40536, USA

**Keywords:** Inflammation, cell polarity, macrophage infiltration, cancer progression, reactive oxygen species

## Abstract

Loss of epithelial cell polarity and inflammation are hallmarks of breast cancer development. Although the association between the disruption of tissue polarity and inflammation has been demonstrated, we know little about how these two events are coupled. Using the 3D co-culture model of mammary epithelial cells and monocytes, a recent study reveals a link between disruption of epithelial polarity and monocytes infiltration.

The establishment and maintenance of epithelial polarity is crucial for the integrity and function of mammary gland. Loss of mammary tissue polarity is associated with cancer development and progression ^[1, 2]^. Disruption of the polarized epithelial tissue structure occurs at the early stage of breast cancer development. This event is associated with the activation of PI3K-Rac1 signaling pathway and the significant remodeling of tissue microenvironment. Inhibition of the PI3K-Rac1 pathway reprograms the non-polarized breast cancer cells to form polarized spheroids in a 3D culture model ^[3, 4]^. Using the 3D culture assay, we found that disruption of polarized acinar structure is accompanied with increased production of reactive oxygen species (ROS) ^[5]^ (Figure 1A). ROS such as hydrogen peroxide, superoxide and the hydroxyl radical, are byproducts of normal metabolism through the electron transport chain. ROS and associated oxidative stress drive cancer development and progression by inducing oxidative damages in DNA, lipids, proteins and other cellular components ^[6, 7]^, but its function and regulation in the disruption of tissue polarity has not been determined.

Treatment with antioxidant agents can reduce ROS levels and reprogram non-polarized breast cancer cells to form polarized spheroids in 3D culture, indicating that elevation of ROS is necessary to disrupt polarized acinar formation. We also found that introduction of a constitutively activated RAC1 is sufficient to induce ROS generation in mammary epithelial cells ^[5]^. Activated RAC1 binds to and forms a complex with NOX1, a homolog of the phagocyte NADPH-oxidase component gp91phox. NOX1 has the capacity to transport electrons across the plasma membrane and to generate superoxide and other downstream ROS. Therefore, RAC1 may increase NOX1-dependent ROS generation. These results suggest that RAC1 is a potential regulator that integrates non-polarized tissue formation and ROS production (Figure 1B).

Macrophages comprise a major stromal component in the tumor microenvironment. The infiltration and differentiation of macrophages determine inflammation in malignant tissue, which in turn promote breast cancer development and progression ^[8, 9]^. Infiltration of tumor-associated macrophages correlates with poor prognosis in breast cancer patients ^[10, 11]^. Macrophage infiltration occurs at an early stage of breast cancer development ^[12, 13]^; therefore, inhibition of early-stage events such as macrophage infiltration and chronic inflammation may offer a promising strategy to prevent or repress cancer progression. However, it remains a challenge to block cancer-associated macrophage infiltration without disturbing normal function of immune system. Using the 3D co-culture model developed in our group, we show that disruption of mammary tissue polarity leads to monocyte/macrophage infiltration during cancer development ^[5]^ . In addition, it has been reported that macrophages accumulate around the terminal end buds of mammary glands rather than near the polarized ductal epithelial cells ^[14, 15]^. Mammary epithelial cells in the terminal end bud are multilayer and non-polarized. These results also suggest that macrophage infiltration is associated with loss of tissue polarity. Interestingly, reducing ROS levels in non-polarized mammary epithelial cells is sufficient to block THP-1 infiltration in 3D culture, indicating that ROS are important mediators of the cancer cell-monocyte interaction (Figure 1B). We show that ROS induce expression of multiple cytokine genes in non-polarized malignant cells ^[5]^. These cytokines may promote recruitment and infiltration of monocytes/macrophages in 3D culture.

The NF-κB pathway is a critical regulator of cytokine expression and macrophage infiltration ^[16]^. The gene expression profile analysis and unbiased position weight matrices analysis (PWMA) ^[17]^ show that the NF-kB pathway is activated in non-polarized mammary epithelial cells ^[18]^. ROS is a well-characterized regulator of the NF-κB pathway. These results suggest the ROS may modulate monocyte/macrophage infiltration by inducing the NF-κB pathway in mammary epithelial cells (Figure 1B). However, how aberrant activation of the NF-κB pathway in mammary epithelial cells induces macrophage infiltration still remains to be addressed.

Given the crucial role of ROS in regulating epithelial cell polarity and macrophage infiltration, reducing ROS levels in mammary epithelial cells may be a promising strategy to inhibit cancer-associate inflammation and prevent cancer development and progression.

## Figures and Tables

**Figure 1:**
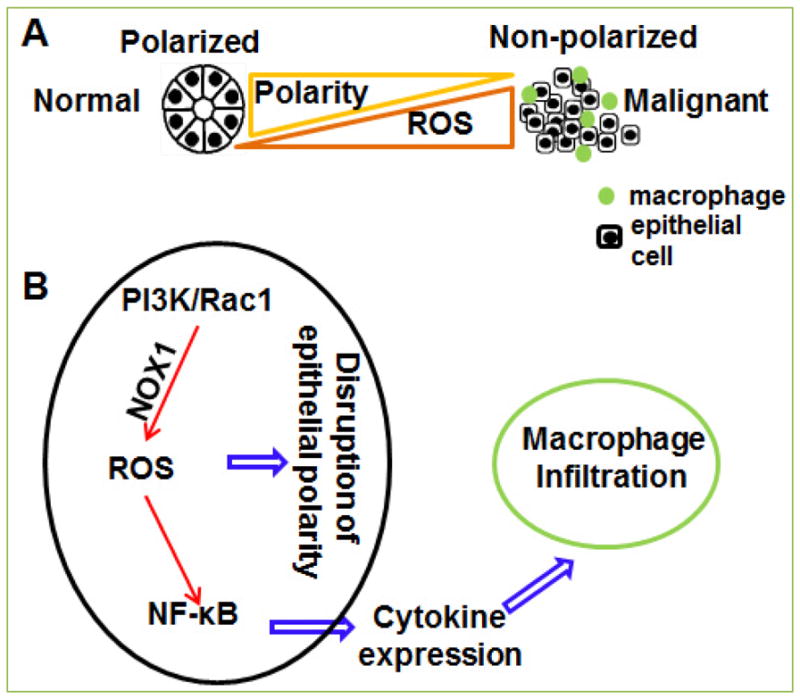
(**A**) **A scheme showing the association of epithelial polarity and ROS production.** (B) Summary overview of the signaling pathway in non-polarized breast cancer cells that induces macrophage infiltration.

## Abbreviations

3D: three-dimensional
ROS: reactive oxygen species
PI3K: Phosphoinositide 3-kinase
PWMA: position weight matrices analysis
